# Factors associated with maternal mortality in eastern Ethiopia: A multicenter case–control study

**DOI:** 10.1002/ijgo.16069

**Published:** 2024-12-07

**Authors:** Mohammed Yuya, Abera Kenay Tura, Sagni Girma, Redwan Ahmed, Marian Knight, Thomas van den Akker

**Affiliations:** ^1^ School of Public Health, College of Health and Medical Science Haramaya University Harar Ethiopia; ^2^ Department of Obstetrics and Gynaecology Leiden University Medical Center Leiden The Netherlands; ^3^ School of Nursing and Midwifery, College of Health and Medical Science Haramaya University Harar Ethiopia; ^4^ Department of Obstetrics and Gynaecology, University Medical Centre Groningen University of Groningen Groningen The Netherlands; ^5^ Department of Obstetrics and Gynaecology Hiwotfana Specialized University Hospital Harar Ethiopia; ^6^ National Perinatal Epidemiology Unit University of Oxford Oxford UK; ^7^ Athena Institute, Vrije Universiteit Amsterdam Amsterdam The Netherlands

**Keywords:** associated factors, audit, Ethiopia, maternal mortality, obstetric complications

## Abstract

**Objective:**

The aim of this study was to identify factors associated with maternal mortality in 13 public hospitals with maternity units in eastern Ethiopia.

**Methods:**

A nested case‐control study embedded within the Ethiopian Obstetric Surveillance System (EthOSS) cohort. EthOSS was established in April 2021 to monitor women admitted with severe obstetric complications (e.g., obstetric hemorrhage, eclampsia, uterine rupture, sepsis, and severe anemia) during pregnancy, childbirth or within 42 days of termination of pregnancy. The cases were all women who died during pregnancy, childbirth, or postpartum in these hospitals, while women who survived these complications were the controls. For each case, we randomly selected three controls. The factors associated with maternal mortality were described using adjusted odds ratios (aOR) with their corresponding 95% confidence intervals (CI). Associations were examined using binary logistic regression analysis followed by multivariable logistic regression analysis for factors with *P* < 0.25. Finally, *P* < 0.05 was considered as the cut‐off for a statistically significant association.

**Results:**

A total of 280 women (70 cases and 210 controls) were included in the study. Compared to survivors, women who died were more likely to have given birth by caesarean section (aOR = 3.35; 95% CI 1.49–7.53), to have been admitted into an intensive care unit (aOR = 6.58; 95% CI 2.08–20.82), to have had postpartum hemorrhage (aOR = 6.39; 95% CI 2.56–15.94), and to have had a pre‐existing medical condition (aOR = 5.39; 95% CI 1.16–24.99).

**Conclusion:**

Improving maternal survival requires appropriate indications for caesarean sections, safe surgical conditions, seamless communication between facilities (particularly in high‐risk pregnancies), adequate multidisciplinary care for women with pre‐existing conditions, and effective intensive care.

## Synopsis

Compared to survivors, women who died had higher odds of having caesarean birth, being admitted into intensive care unit, having postpartum hemorrhage and having pre‐existing medical condition.

## 1 INTRODUCTION

Reducing maternal mortality remains an unfinished global agenda item. In 2020, 287 000 maternal deaths (800 per day) occurred worldwide, from which 70% were in sub‐Saharan Africa (SSA). SSA had the highest maternal mortality ratio (MMR) in 2020, estimated at 545 per 100 000 livebirths, compared to the global MMR of 223 per 100 000.^1^ While the MMR in Ethiopia, 267 per 100 000 live births in 2020, indicated a reduction from the MMR of 401 per 100 000 in 2017,^2^ Ethiopia still had the highest absolute number of maternal deaths (10 000) next to Nigeria, India, and the Democratic Republic of the Congo.^1^, ^2^

Several initiatives were started to reduce maternal mortality in Ethiopia, from introducing programs such as the maternal death surveillance and response (MDSR) program in 2013 to training a larger number of health professionals and expanding the number of emergency obstetric care facilities.^3^, ^4^, ^5^, ^6^ However, hemorrhage, hypertensive disorders of pregnancy, and sepsis continue to be prevalent causes of maternal mortality.^3^

Research efforts have so far mainly focused on describing causes of deaths and have infrequently presented any comparisons with women who survived particular complications. This is also true for the annual MDSR reports, which focused only on women who died and causes of deaths, without considering women who survived severe complications.^3^, ^7^, ^8^ This limited scope hampers the identification of factors associated with mortality in the pathophysiological pathway and the designing of contextualized interventions.^9^, ^10^, ^11^

The 2030 target of reducing the global MMR to below 70 per 100 000 livebirths, with no country having more than 140 per 100 000 live births, requires understanding of basic determinants of pathways to mortality to design tailored interventions.^12^ In this study, we assessed the factors associated with maternal mortality among women who sustained a pre‐defined set of maternal morbidities, by comparing women who died with those who survived obstetric complications in eastern Ethiopia.

## 2 MATERIALS AND METHODS

### 2.1 Data sources

This study was conducted as part of the Ethiopian Obstetric Surveillance System (EthOSS).^6^ EthOSS is a prospective facility‐based regional surveillance of major adverse obstetric conditions (i.e., obstetric hemorrhage, eclampsia, uterine rupture, sepsis, and severe anemia) in maternity units of 13 public health hospitals in Harari Region, Dire Dawa City Administration, East and West Hararghe Zones of Oromia Region, Eastern Ethiopia. These public hospitals range from district hospitals providing comprehensive emergency obstetric care to tertiary academic centers with an advanced level of care and diagnostic services. Details of EthOSS methodology are described elsewhere.^6^ In brief, all maternal deaths and women with any of the five adverse obstetric conditions were reported on a monthly basis by a designated midwife in each hospital. After receiving the reports, EthOSS data collectors visited respective hospitals and reviewed each case for eligibility and collected detailed information.

### 2.2 Study design and population

This was a nested case–control study embedded within the EthOSS cohort. Cases were all women who died during pregnancy or childbirth or within 42 days of termination of pregnancy in the 13 hospitals from April 1, 2021 to 31 March 2022. Women who were admitted to the respective hospitals during the study period for any of the major adverse obstetric conditions but survived were the controls. We included all maternal deaths during the study period. For each case, three controls were randomly selected from the EthOSS database using Stata 16.

### 2.3 Data collection

At each hospital, a designated midwife reported the number of maternal deaths and women with any of the EthOSS conditions (obstetric hemorrhage, eclampsia, uterine rupture, sepsis, and severe anemia) on a monthly basis. As described elsewhere, obstetric hemorrhage was defined as excessive bleeding (usually related to pregnancy) in parturient. The definition included both antepartum and postpartum hemorrhage. Antepartum hemorrhage included severe bleeding from or into the genital tract, occurring from 28 + 0 weeks of pregnancy and prior to the birth of the baby while postpartum hemorrhage referred to excessive bleeding (more than 500 mL for vaginal delivery and 1000 mL for caesarean delivery) following the birth of a baby or a drop in hematocrit of >10% from baseline. Eclampsia was defined as diastolic blood pressure ≥90 mm Hg or proteinuria +3 and presence of convulsions or coma. Similarly, uterine rupture was defined as complete rupture of the uterus during labor, confirmed by laparotomy or autopsy. Sepsis was defined as a clinical suspicion of infection and three of the following: temperature >38°C, respiration rate <20/min, pulse rate >90/min, or WBC >12 000. Severe anemia was defined by a hemoglobin level of <7 mg/dL. Pre‐existing medical conditions was defined as a conditions or illnesses that the woman has before becoming pregnant, such as cardiac disease, diabetes mellitus, hypertension, tuberculosis, HIV, or renal disorders.^6^, ^13^

The EthOSS protocol was reviewed and approved by the Institutional Health Research Ethics Review Committee (Ref No. IHRERC/024/2021) of the College of health and medical Sciences of Haramaya University, Ethiopia and the University of Oxford's Oxford Tropical Research Ethics Committee (OxTREC Reference 530‐21). Before the actual data collection, informed consent was obtained from the head of each hospital.

Once the report was submitted to EthOSS, EthOSS data collectors were dispatched to collect detailed information about each reported woman after checking for eligibility. Six trained research assistants collected the data online using the Kobo toolbox.^14^ In addition, data on the number of births per month, number of livebirths, and admissions in the maternity units were obtained from the hospital Health Management Information System (HMIS) database. All collected data were anonymously stored on a secure system. The overall data collection was supervised by SG, RA, and AKT.

### 2.4 Statistical analysis

**FIGURE 1:**
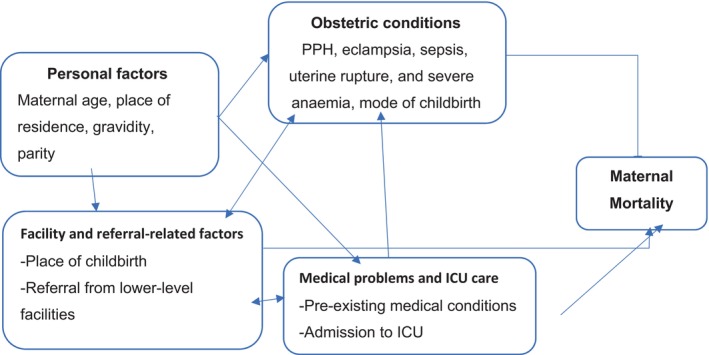
Directed acyclic graph for factors associated with maternal mortality in Eastern Ethiopia.

After ensuring completeness and consistency, data were exported to Stata 16 for analysis. Variables such as educational status, occupational status, monthly income, and marital status, which are less documented on women's files, were excluded. Descriptive statistics were used to describe findings of socio‐demographic and pregnancy‐related characteristics using tables and graphs with frequencies and percentages. Guided by a directed acyclic graph (DAG) (Figure 1), associations between dependent and independent variables were examined using crude odds ratios (cOR) during binary logistic regression analysis, followed by multivariable logistic regression analysis for those associated with a *P* < 0.25. We used the adjusted odds ratio (aOR) along with its 95% confidence level (CI) to measure the strength of the associations; *P* < 0.05 was considered as the cut‐off for a statistically significant association. The multicollinearity was assessed to check if there is any correlation between independent variables.

## 3 RESULTS

### 3.1 Socio‐demographic and obstetric characteristics of participants

A total of 70 women who died and 210 controls were included. Most cases (50, 71.43%) and controls (157, 75.85%) were 20–34 years old, and (46, 65.71%) and (140, 66.99%) of the cases and controls were from rural areas. Cases and controls had similar characteristics, except for location of birth, referral status, and having postpartum hemorrhage and pre‐existing medical conditions (Table 1).

**TABLE 1:** Socio‐demographic and obstetric characteristics of women admitted with severe obstetric complications at public hospitals in Eastern Ethiopia (*n* = 280, cases = 70, and controls = 210).

Characteristics	Deaths, *N* (%)	Survivors, *N* (%)	*P*‐value^a^
Maternal age (years)			
<20	4 (5.71)	23 (11.11)	0.084
20–34	50 (71.43)	157 (75.85)	
≥35	16 (22.86)	27 (13.04)	
Place of residence			
Rural	46 (65.71)	140 (66.99)	0.329
Urban	24 (34.29)	69 (33.01)	
Hospital type			
Primary hospital	8 (21.05)	30 (78.95)	0.610
General hospital	39 (27.46)	103 (72.54)	
Tertiary hospital	23 (23.00)	77 (77.00)	
Parity			
1	10 (19.23)	22 (15.94)	0.849
2–4	21 (40.38)	60 (43.48)	
>4	21 (40.38)	56 (40.58)	
Attended antenatal care			
Yes	21 (31.82)	68 (32.85)	0.876
No	45 (68.18)	139 (67.15)	
Any pre‐existing medical condition			
Yes	6 (8.69)	5 (2.39)	0.020
No	63 (91.30)	204 (97.61)	
Referred from other facilities			
Yes	53 (77.94)	103 (49.05)	0.000
No	15 (22.06)	107 (50.95)	
Mode of birth			
Caesarean	21 (42.00)	36 (20.69)	0.002
Vaginal (including instrumental)	29 (58.00)	138 (79.31)	
Place of birth			
Home	9 (17.31)	22 (12.64)	0.391
Health facility	43 (82.69)	152 (87.36)	
Admitted to intensive care unit			
Yes	13 (19.40)	11 (5.26)	0.000
No	54 (80.60)	198 (94.74)	
Anemia (Hemoglobin <7 mg/dL)			
Yes	18 (31.03)	56 (28.87)	0.750
No	40 (68.97)	138 (71.13)	
Had postpartum hemorrhage			
Yes	19 (27.14)	18 (8.57)	0.000
No	51 (72.86)	192 (91.43)	
Had eclampsia			
Yes	20 (28.57)	47 (22.38)	0.293
No	50 (71.43)	163 (77.62)	
Had sepsis			
Yes	8 (11.43)	37 (17.62)	0.21
No	62 (88.57)	173 (82.38)	

^a^ χ^2^.

### 3.2 Distribution of cases and controls in 13 public hospitals under Ethiopian Obstetric Surveillance System Consortium

**FIGURE 2:**
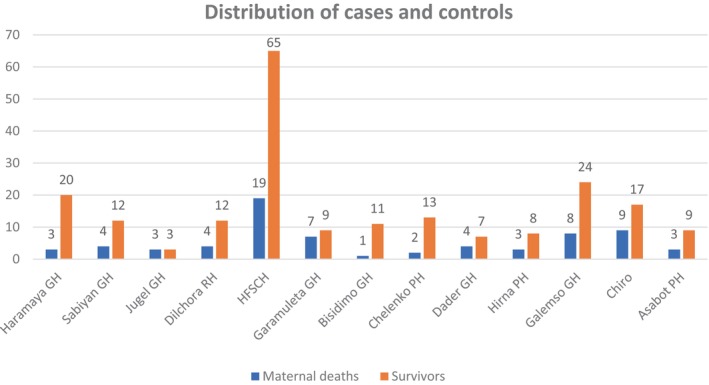
Distribution of cases and controls in 13 public hospitals under Ethiopian Obstetric Surveillance System (EthOSS) consortium in Eastern Ethiopia (*n* = 280, cases = 70, and controls = 210).

Based on the catchment areas and type of the hospital, the distribution of cases and controls were different. Accordingly, more than one‐quarter 19 (27.14%) of cases were from Hiwot Fana Specialized Comprehensive Hospital (HFSCH), the only tertiary hospital in eastern Ethiopia, followed by nine (12.86%) of cases from Chiro GH, eight (11.43%) of cases from Gelemso GH, seven (10.00%) of cases from Garamuleta GH, and less than five cases were reported from other hospitals. The controls were also high (65, 30.95%) in HFSCH, followed by 24 (11.43%) in Gelemso GH, 20 (9.52%) in Haramaya GH, and 17 (8.09%) in Chiro GH, and fewer than 13 (6.19%) controls in others hospital were reported (Figure 2).

### 3.3 Case fatality rate of Ethiopian Obstetric Surveillance System Consortium condition

**FIGURE 3:**
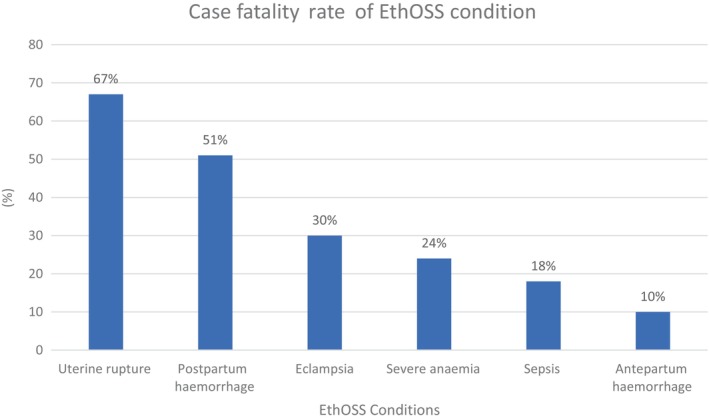
Case fatality rate of Ethiopian Obstetric Surveillance System (EthOSS) conditions among women admitted with severe obstetric complications at public hospitals in Eastern Ethiopia.

The proportions of women who died from uterine rupture, postpartum hemorrhage, eclampsia, severe anemia, sepsis, and antepartum hemorrhage were 10 (66.67%), 19 (51.35%), 20 (29.85%), 18 (24.32%), 8 (17.78%), and 5 (9.26%), respectively (Figure 3).

### 3.4 Factors associated with maternal mortality

After adjusting for potential confounders, compared to their counterparts, women who died were more likely to have given birth by caesarean section (aOR = 3.35; 95% CI 1.49–7.53), to have been admitted to an intensive care unit (aOR = 6.58; 95% CI 2.08–20.82), to have postpartum hemorrhage (aOR = 6.39; 95% CI 2.56–15.94), and to have had a pre‐existing medical condition (aOR = 5.39; 95% CI 1.16–24.99) (Table 2).

**TABLE 2:** Factors associated with maternal mortality in public hospitals in Eastern Ethiopia (*n* = 280, cases = 70, and controls = 210).

Variables	cOR, 95% CI	aOR, 95% CI	*P*‐value
Maternal age			
<20 years	1	1	
20–34 years	1.83 (0.60, 5.54)	6.09 (0.61, 61.13)	0.124
≥35 years	3.41 (0.99, 11.64)	9.61 (0.87, 105.87)	0.065
Place of residence			
Rural	1.34 (0.74, 2.42)		
Urban	1		
Hospital type			
Primary hospital	1		
General hospital	1.42 (0.60, 3.36)		
Tertiary or Referral hospital	1.12 (0.45, 2.78)		
Parity			
1	1		
2–5	0.76 (0.57, 2.28)		
>5	0.87 (0.66, 2.58)		
Had pre‐existing medical condition			
Yes	3.88 (1.15, 13.16)	5.39 (1.16, 24.99)	0.031
No	1	1	
Referred from other facility			
Yes	3.67 (1.95, 6.92)	1.92 (0.87, 4.19)	0.102
No	1	1	
Place of birth			
Home	1.45 (0.62, 3.37)		
Health facility	1		
Mode of birth			
Caesarean	2.77 (1.42, 5.43)	3.35 (1.49, 7.53)	0.003
Vaginal	1	1	
Had postpartum hemorrhage			
Yes	3.97 (1.94, 8.12)	6.39 (2.56, 15.94)	0.000
No	1	1	
Admitted to intensive care unit (ICU)			
Yes	4.33 (1.84, 10.21)	6.58 (2.08, 20.82)	0.001
No	1	1	
Had eclampsia			
Yes	1.38 (0.75, 2.56)		
No	1		
Had sepsis			
Yes	0.60 (0.27, 1.37)		
No	1		
Severe anemia (<7 mg/dL)			
Yes	1.11 (0.59, 2.09)		
No	1		

Abbreviations: aOR, adjusted odds ratio; cOR, crude odds ratio.

## 4 DISCUSSION

### 4.1 Overall findings

In this study, we identified factors associated with maternal mortality among women with severe obstetric complications using a multicenter regional facility‐based surveillance study as part of EthOSS. We found that having pre‐existing medical conditions, caesarean birth, postpartum hemorrhage, and intensive care unit admission were associated with increased odds of maternal mortality. Our study is the first of its kind to describe factors associated with maternal mortality among women admitted with major obstetrics complications using multicenter prospective data collection in Ethiopia.

Despite availability of evidence‐based options for prevention and management, it is concerning that uterine rupture and PPH continue to be major causes of maternal mortality. While the importance of addressing uterine rupture and PPH is well recognized by the Ministry of Health, and a call to action to halt deaths from these conditions has been in place since 2005,^15^ much more effort is still needed to achieve this goal. Beyond awareness creation, which is common practice during celebration of the Safe Motherhood Month by the Ethiopian Ministry of Health, implementing appropriate and effective interventions is essential. Criterion‐based audit, safe caesarean section services at lower‐level health facilities, establishing a blood bank at all CEmONC facilities, and comprehensive PPH treatment bundles were reported to be effective.^16^, ^17^, ^18^

Compared to women who survived obstetric complications, women who died were more likely to give birth by caesarean section, as confirmed by a previous study in Ethiopia.^19^ Studies conducted in Brazil and the Netherlands indicated a threefold increase in maternal deaths among women who gave birth by caesarean section.^20^, ^21^, ^22^ This might be related to the complications leading up to the caesarean section, absence of safe surgical practice, or perioperative care including ICU care. Other studies also report the impact of caesarean section on maternal mortality, including a systematic review and meta‐analysis in low and middle income countries.^23^ Whether a death is due to the caesarean section itself or whether the latter was performed as a life‐saving intervention in a woman already in critical condition requires a different analysis. The decision for caesarean section should be made by an experienced clinician, and the procedure performed by someone with appropriate surgical skills. Both conditions are not always met.^24^, ^25^, ^26^

In this study, more than one third of women who died were admitted for ICU care but more than half of those admitted to ICU died. This is likely to be related to the severity of cases, but there also is a need to improve ICU care. This is in line with a previous study from the capital Addis Ababa,^27^ and multi‐country surveys.^28^, ^29^ The majority of ICUs in these hospitals lack essential lifesaving equipment like mechanical ventilators and dialysis.

Consistent with previous studies,^5^, ^30^ more women who died had pre‐existing medical conditions. Low levels of antenatal care (with limited screening and content) and preconception care might contribute to this problem,^31^, ^32^ and strengthening antenatal and preconception care is essential to improve maternal care.

### 4.2 Implications of the study findings

While evidence on why women die is not new, the continued deaths of women from causes we are familiar with requires a paradigm shift if the 2030 Sustainable Development Goals are to be met. In addition to simply recommending ‘interventions’, such as the strategies proposed in the national ‘Ending Preventable Maternal Mortality’ initiative,^12^ there is a need for context‐specific implementation, including co‐creation and co‐owning. Recently, evidence from co‐creation of context‐specific interventions in maternal and child health has been gaining more attention. While the impact of such interventions on maternal mortality is yet to be studied, its significance in improving intrapartum care and perinatal outcomes has already been documented in Zanzibar and elsewhere in Tanzania.^33^, ^34^, ^35^ Given the high burden of severe maternal outcomes in eastern Ethiopia,^6^ a plan for improving care through co‐creation of interventions and using low‐dose high‐frequency training and seminars is planned in all EthOSS hospitals following the PartoMa approach.^33^, ^36^

### 4.3 Strengths and limitations

A major strength of the study is that all hospitals with maternity units in eastern Ethiopia were included. Prospective identification and registration of women enabled us to comprehensively capture cases by designated midwives, which is thought to minimize under‐reporting. Although identification of women with these conditions was facilitated through prospective registration, we had to rely on paper‐based medical records with sometimes incomplete information. Given that this study was hospital‐based, women who were treated in lower‐level facilities or who remained at home might have been missed.

## 5 CONCLUSION

Overall, women who died were more likely to have had postpartum hemorrhage, to have given birth by caesarean section, to have been admitted to ICU, or to have had pre‐existing medical conditions. A case‐by‐case audit of management would be required to analyze quality of care in detail, including timeliness of interventions. In addition, interdepartmental communication for women with high‐risk pregnancies, appropriate indications for caesarean section, advanced ICU and perioperative care, and a functional blood bank are lifesaving.

## AUTHOR CONTRIBUTIONS

Abera Kenay Tura, Thomas van den Akker, and Marian Knight designed the study. Mohammed Yuya and Abera Kenay Tura undertook the analysis. Mohammed Yuya drafted the first manuscript with close support from Abera Kenay Tura. Abera Kenay Tura, Sagni Girma, Redwan Ahmed, Thomas van den Akker, and Marian Knight reviewed the manuscript for intellectual content and edited and revised the manuscript. All authors read and approved the final manuscript for submission.

## FUNDING INFORMATION

The study was funded by MRC (MR/T037962/1) as part of the 2019 Global Maternal and Neonatal Health Funding call. MY is funded by Leiden University Medical Center for his PhD study. MK is a National Institute for Health and Care Research (NIHR) senior investigator. The funders had no role in the study design, data collection and analysis, manuscript preparation, or the decision for publication. The views expressed in this publication are those of the author(s) and not necessarily those of the NHS, NIHR, or the Department of Health and Social Care.

## CONFLICT OF INTEREST STATEMENT

None.

## Data Availability

All data relevant to this article are included in the manuscript. The aggregate de‐identified data used for this study are available based on reasonable request to AKT.
